# Detection of West Nile virus in six mosquito species in synchrony with seroconversion among sentinel chickens in India

**DOI:** 10.1186/s13071-016-1948-9

**Published:** 2017-01-07

**Authors:** Siraj A. Khan, Purvita Chowdhury, Parveena Choudhury, Prafulla Dutta

**Affiliations:** Regional Medical Research Centre, NE Region, ICMR, Dibrugarh, 786001 Assam India

**Keywords:** West Nile virus, Lineage 5, Mosquito vectors, *Culex pseudovishnui*, *Mansonia uniformis*, Sentinel chickens

## Abstract

**Background:**

West Nile virus (WNV) is a zoonotic flavivirus maintained in mosquito-bird transmission cycle. Although humans are accidental hosts, fatal outcomes following WNV infection have been reported from India. Studies have identified WNV as an important etiological agent causing acute encephalitis syndrome in Assam, Northeast India. While circulation of WNV is evident, the role of vectors and avian hosts involved in the transmission remains unclear. In this study we identified local mosquito species for evidence of WNV infection along with seroconversion among sentinel chickens.

**Methods:**

Mosquitoes were collected and pooled species wise from June 2014 through December 2015. Virus was screened using reverse transcriptase PCR followed by sequencing and phylogenetic analysis. Sentinel chicken blood was screened for WNV antibody to assess their role in WNV transmission.

**Results:**

A total of 52,882 mosquitoes belonging to 16 species were collected. WNV was detected in 18 pools of *Culex vishnui*, *Culex tritaeniorhynchus*, *Culex quinquefasciatus*, *Culex whitmorei*, *Culex pseudovishnui* and *Mansonia uniformis.* Phylogenetic analysis revealed that all mosquito derived sequences belonged to Lineage 5 and were 99–100% similar to the Assam strain of WNV isolated from human CSF sample in 2007. All sentinel chickens had seroconverted by the month of July that happens to be the peak WNV transmission month among humans as well.

**Conclusion:**

To the best of our knowledge, this is the first report of WNV identification from field-collected *Cx. pseudovishnui* and *Mansonia uniformis* in India. Our study demonstrates potential vectors which may play a crucial role in WNV transmission and should be considered in the vector control strategies. Additionally, our study highlights the role of sentinel chickens for WNV surveillance.

**Electronic supplementary material:**

The online version of this article (doi:10.1186/s13071-016-1948-9) contains supplementary material, which is available to authorized users.

## Background

West Nile virus (WNV) has emerged as one of the most widespread flavivirus being reported from all the continents except Antarctica [1]. Since its first outbreak in 1937, the disease attained immense attention during the mid-1990s corresponding to its severity, frequency and geographical expansion [2–4].

WNV is maintained naturally in an enzootic transmission cycle of mosquitoes (vectors) and birds (amplifying hosts) while humans, horses and other mammals serve as accidental/dead end hosts. Among a number of mosquitoes involved in epizootic and epidemic transmission including species of *Anopheles* (in USA, Isreal, Madagascar), *Aedes* (in Africa, Russia, USA), *Mansonia* (Africa) and *Ochlerotatus* (USA)*,* the most important vectors of WNV represent *Culex* spp. [5]*.* In India, WNV has been isolated from *Cx. vishnui*, *Cx. quinquefasciatus*, *Cx. tritaeniorhynchus* and *Cx. fatigans* [6]. House sparrows and corvids have been implicated as important WNV reservoirs in North America, Europe and Africa. However, in the Indian subcontinent, ardeid birds are thought to be the possible amplifying hosts [7].

WNV infection in humans can induce symptoms ranging from febrile fever to severe neurological syndromes like encephalitis, meningitis and paralysis [8]. In India, WNV-neutralizing antibodies were first detected in 1952 [9]. Since then, the virus has been isolated from different hosts and regions of the country. Recognition of WNV among acute encephalitis syndrome causing etiologies in Assam in 2006 was the first report of the flavivirus from eastern region of India [10]. Genetic characterization of two WNV isolates obtained from this region revealed similarity with south Indian WNV Lineage 5 strains [11]. Although WNV circulation in this region is evident, adequate knowledge of vectors and amplifying hosts involved in transmission of WNV are lacking. In this study we investigated local mosquito species as candidate WNV vectors along with the role of birds as amplifying hosts.

## Methods

### Study sites

**Fig. 1:**
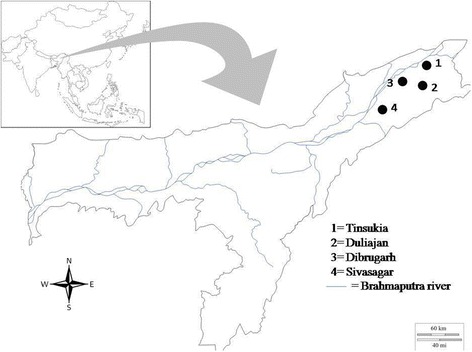
Locations of mosquito collection in Assam, India. Mosquitoes were collected fortnightly at the four sites from June 2014 through December 2015

Study sites were selected based on maximum number and frequency of WN cases reported during earlier outbreaks in the eastern regions of the State of Assam. Adult mosquitoes were collected from four townships: Dibrugarh (27.4728°N, 94.9120°E); Tinsukia (27.4922°N, 95.3468°E); Sivasagar (26.9826°N, 94.6425°E); and Duliajan (27.3572°N, 95.3223°E) (Fig. 1). The sites were kept unchanged throughout the study. The selected townships have numerous water bodies that serve as abodes for migratory birds during winters.

### Entomological study

Mosquito collection was carried out for 1–2 h during dusk using mechanical aspirators at fortnightly intervals from June 2014 through December 2015. Mostly cattle sheds were targeted which are open on three sides with a close proximity to human dwellings. All mosquitoes were identified following standard entomological keys [12–14] under immobilization (by cold shock) and pooled species wise for incrimination studies. Although we did not calculate the proportionate ratio, it comprised of non-fed, partially-fed as well as fully-fed mosquitoes. However, the fully-fed ones were kept in the insectarium for over 24 h for digestion of the blood meal before identification and pooling. Species wise mosquito man hour density (MHD) was calculated as

MHD = number of mosquitoes collected × 60/time spent in minutes × number of persons involved in collections.

### Molecular analysis

Every mosquito pool was triturated mechanically using 2% foetal bovine serum (FBS) (Gibco, Thermo Fisher Scientific, Massachusetts, USA) in pre chilled minimum essential medium (MEM) (Sigma-Aldrich, St. Louis, MO, USA) treated with 50 U penicillin (Sigma-Aldrich, St. Louis, MO, USA), 50 μg/ml streptomycin (Sigma-Aldrich, St. Louis, MO, USA), and 50 μg/ml amphotericin B (Sigma-Aldrich, St. Louis, MO, USA). The homogenate after centrifugation was processed for RNA extraction as per manufacturer’s instructions (QIAamp Viral RNA Kit, Qiagen, Germany).

Presence of WNV RNA was screened in all the mosquito pools using a semi-nested reverse transcriptase (RT)-PCR performed on verity 96 well thermal cycler (Applied Biosystems, California, USA). Primers used for WNV RNA amplification have been described by Khan et al. [15] giving a 500 bp product. Briefly, the first round of amplification was performed with 2 μl of re-suspended RNA template using Access one step RT-PCR kit (Promega, Wisconsin, USA). The first PCR amplicon (1 μl) was then subjected to semi nested PCR using 2× Master Mix (Promega, Wisconsin, USA) with 200nM of reverse primer (sn5'-TGG CCA AGA ACA CGA CCA GAA GG-3') in a final volume of 15 μl. PCR profile for the second round of amplification were carried out by denaturing at 94 °C for 5 min, followed by 35 cycles of 94 °C for 30 s, 54 °C for 1 min and 72 °C for 1 min and final extension at 72 °C for 7 min. All positive amplicons were confirmed by sequencing commercially (Avantor, Selangor, Malaysia).

### Phylogenetic analysis

The resulting forward and reverse sequences were manually edited in BioEdit version 7.0.9 software [16]. The edited nucleotide sequences were compared with a total of 20 WNV GenBank sequences which were selected on the basis of their lineage and geographical origin for determination of WNV lineage. A GenBank sequence (AF080251) of Indian Japanese encephalitis virus (JEV) strain was used as the out-group. Phylogenetic analysis was carried out using Mega 7 software [17]. The Clustal W program implemented in Mega 7 software was used to generate a multiple alignment of the sequences and subsequently construct character based maximum likelihood (ML) tree. Nucleotide and amino acid sequence similarity was estimated in Mega 7 using the gamma distribution (shape parameter = 5). Reliability of the tree was estimated by 1000 bootstrap replications.

### Avian study

In cooperation with the Forest Department, Government of Assam, India, a strict vigilance on any unusual mortality of birds in the reserve forests and national parks of Assam was maintained. However, no such incidence (either among migratory or local birds) was reported during the study period. Subsequently, we attempted to look for WNV seroconversion in sentinel chickens to ascertain the role of domestic birds in WNV transmission as circulation of WNV has been evident among humans in this region for the past decade [10]. Sentinel chickens with each flock consisting of 10 birds were established at two of the study sites, Dibrugarh and Sivasagar, during April 2015 - August 2015. Blood samples were collected fortnightly by using brachial venipuncture method, and the separated serum was stored at -80 °C until serological tests were performed.

### Serological analysis

To detect WNV-specific antibodies, chicken serum samples were tested in 96 well microtitre plates using hemagglutination inhibition (HI) test [18]. Briefly, removal of nonspecific inhibitors in chicken sera was achieved by acetone extraction. Acetone treated chicken sera were serially diluted and mixed with four hemagglutination (HA) units of WNV antigen. Following an overnight incubation at 4 °C, goose red blood corpuscles (RBCs) were added and the solution was incubated at room temperature for another hour. The HI titre was expressed as the highest serum dilution producing complete inhibition of RBC agglutination. Sera with HI titre of 1:10 were considered as positive. Due to cross-reactivity among JEV and WNV in endemic region, the chicken sera were also tested against JEV antigen. Consequently, WNV antibodies were distinguished by comparing both WNV and JEV HI titres [19].

### Statistical analysis

The minimum infection rate (MIR) was calculated as the number of infected mosquitoes per 1000 mosquitoes tested [20]. Differences in chicken seroprevalence rates among the two study sites were tested by unpaired Student’s *t*-test. The temporal concordance of sentinel chicken seroconversion and mosquito MHD was evaluated by cross correlation analysis.

## Results

### Mosquito samples and viral detection

During June 2014 - December 2015, a total of 52,882 adult mosquitoes belonging to 16 species (including 10 known potential vectors of WNV) were collected and analysed. Our collections showed an almost equal distribution of mosquito density in all the study sites and the total number of samples per site is given in Table 1. *Mansonia uniformis*, *Culex vishnui* and *Culex tritaeniorhynchus* were the predominant species constituting 26.32, 24.47 and 13.50% of the total catch, respectively. Eighteen of the 1392 mosquito pools tested RT-PCR positive for WNV non-structural 1 (NS1) gene. Majority of the positive pools consisted of *Ma. uniformis* and *Cx. vishnui* followed by *Cx. tritaeniorhynchus*, *Cx. quinquefasciatus*, *Cx. pseudovishnui* and *Cx. whitmorei* (Table 2). The MIR of *Cx. whitmorei* was found to be highest.

**Table 1:** Distribution of adult mosquitoes of sixteen species collected at the four study sites

Species/ Study site	Dibrugarh	Tinsukia	Duliajan	Sivasagar
*Aedes albopictus* ^a^	4	430	0	0
*Aedes nigrostriatus*	2	29	28	73
*Aedes. vexans* ^a^	2	64	0	0
*Anopheles annularis*	124	175	60	319
*Anopheles hyrcanus* ^a^	480	114	379	639
*Anopheles vagus*	137	35	50	114
*Armigeres kuchingensis*	350	283	308	242
*Culex fuscocephala*	34	65	150	163
*Culex gelidus* ^a^	0	0	70	7
*Culex pseudovishnui* ^a^	1453	1512	1365	1147
*Culex quinquefasciatus* ^a^	1380	838	1540	262
*Culex tritaeniorhynchus* ^a^	1925	1573	1816	1822
*Culex vishnui* ^a^	3033	3413	4027	2470
*Culex whitmorei* ^a^	599	1598	500	404
*Mansonia annulifera*	136	649	226	342
*Mansonia uniformis* ^a^	2819	2566	4509	4028
Total	12,478	13,344	15,028	12,032

^a^Potential WNV vector

**Table 2:** Mosquito species found to be WNV-positive by RT-PCR and sequencing from the four study sites

Species	Dibrugarh	Tinsukia	Duliajan	Sivasagar	MIR
	Positive/Pools tested	Positive/Pools tested	Positive/Pools tested	Positive/Pools tested	
*Cx. pseudovishnui*	0/36	0/27	0/35	2/29	0.36
*Cx. quinquefasciatus*	2/44	0/30	0/41	0/13	0.49
*Cx. tritaeniorhynchus*	1/44	1/38	1/46	0/41	0.42
*Cx. vishnui*	0/67	1/90	0/85	4/57	0.38
*Cx. whitmorei*	1/21	0/47	0/21	0/15	0.55
*Ma. uniformis*	2/71	0/66	2/101	1/89	0.35

*Abbreviation*: *MIR* minimum infection rate; calculated as (number of positive pools/total number of specimen tested) × 1000

### Phylogenetic analysis

**Fig. 2:**
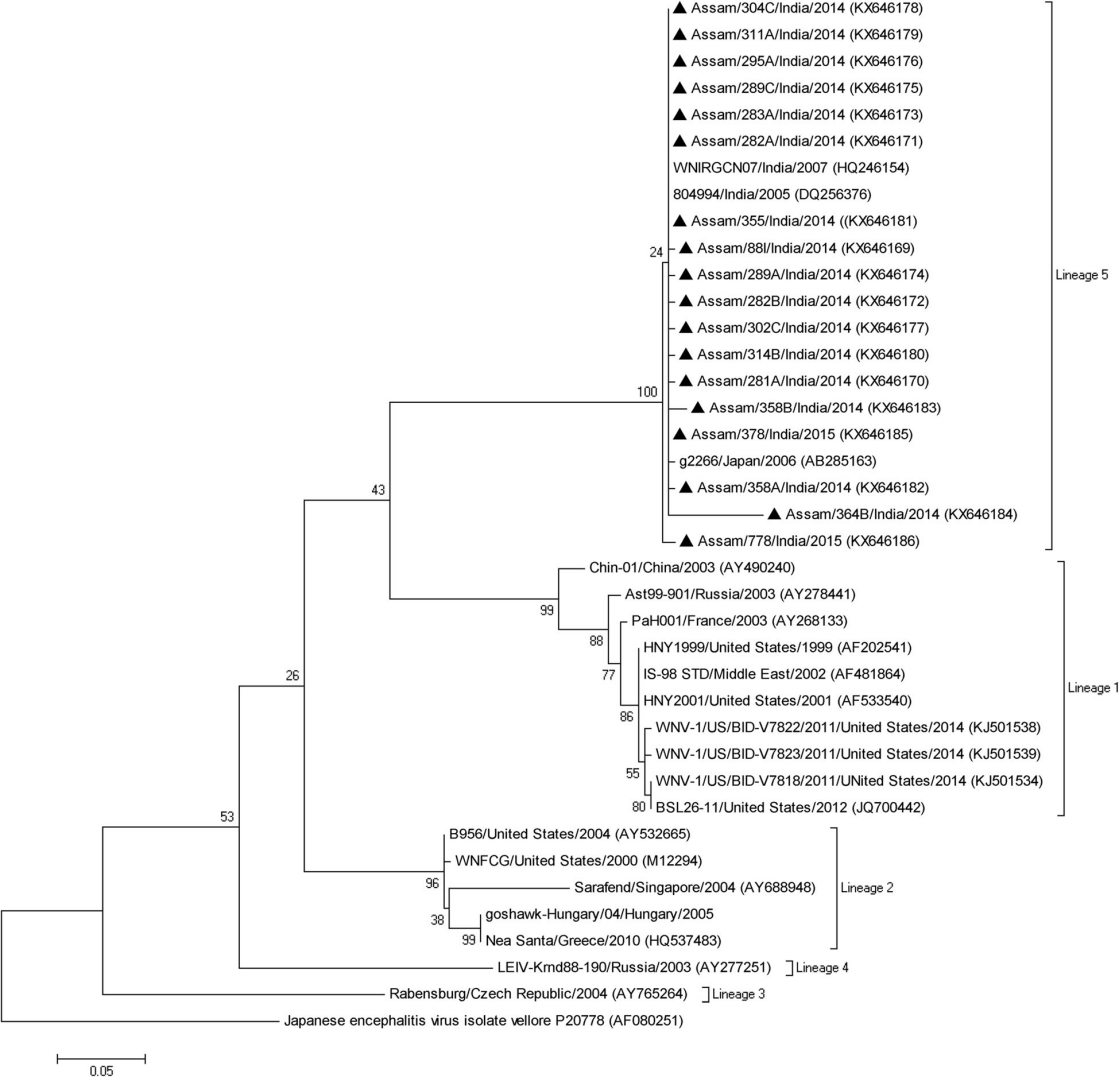
Phylogenetic analysis of geographically distinct West Nile virus sequences based on 500 bp region of the NS1 gene. Black triangles denote the mosquito-derived sequences from this study. The maximum likelihood tree was constructed using Kimura 2-parameter model in Mega 7. An Indian strain (P-20778) of Japanese encephalitis virus (GenBank accession no. AF080251) was used as an outgroup. Node values were estimated for 1000 replicates

Partial sequence of WNV NS1 region of *.*500 bp was obtained from 18 mosquito pools (GenBank Accession nos. KX646169–KX646186). The derived phylogenetic tree revealed that the sequences obtained during the study formed a clade within the Lineage 5 in the tree (Fig. 2). Furthermore, it indicated that the circulating WNV in Assam during 2014–2015 was similar to the Lineage 5 strain isolated in Assam during 2007 (HQ246154). An additional file shows that all the WNV positive sequences were more than 99% similar to the 2007 Assam isolate (see Additional file 1: Table S1). The mean genetic distance of 0.01% was found at the nucleotide level. On alignment of each mosquito-derived sequence with 2005 Indian strain (DQ256376) and 2007 Assam strain (HQ246154), a few synonymous and non-synonymous mutations were observed. Interestingly, two synonymous mutations, i.e. C → T and T → G, were found in all mosquito sequences except for KX646169. Moreover, two nucleotide substitutions resulted in changes in amino acid codons, i.e. from valine to alanine in three mosquito-derived sequences. It is worth mentioning that one of the sequences (KX646186) formed a distinct sub-clade pertaining to a few synonymous and non-synonymous substitutions.

### Serological analysis

**Fig. 3:**
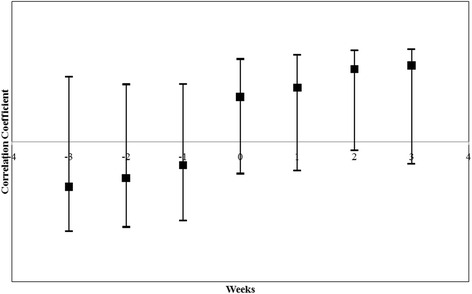
Relationship between *Ma. uniformis* MHD and lagged sentinel chicken seroconversion (lead and lag shown on the x-axis) represented by correlation coefficient with 95% confidence interval

It was observed that 70% of the chicken sera had higher WNV compared to JEV HI titer. Seroconversion in sentinel chickens started towards end of May and antibodies against WNV were detected in all sentinel chicken by July 2015. Significant differences in seroprevalence rates among the two study sites were not observed in the present study. The percentage of chicken seroconversion during May to August 2015 was compared with the abundance of incriminated WNV vectors. Chicken seroconversion peak coincided with the increase in abundance of three incriminated WNV vectors, *Ma. uniformis*, *Cx. vishnui* and *Cx. whitmorei.* However, peaks in abundance of *Cx. pseudovishnui* and *Cx. tritaeniorhynchus* were preceded by peaks in chicken seroconversion. The cross correlation with 95% CI revealed that sentinel chicken seroconversion (*r* = 0.77, *P* = 0.06) was marginally associated to the density of *Ma. uniformis* mosquito after 2 weeks (Fig. 3).

## Discussion

The present study detected WNV belonging to Lineage 5 from six species of mosquitoes and corroborated that the transmission of WNV involves bird-mosquito cycle in upper basins of the Brahmaputra valley of Assam, Northeast India. The geographical distribution and abundance of potential WNV vectors at all of the four study sites suggests the possibility of active circulation of WNV. Cattle sheds targeted during mosquito collection harbor abundant zoophilic mosquito species. As almost all the potential/incriminated vector species are exophilic and exophagic in nature, it is unlikely that important mosquito species could have been missed. As anticipated, *Ma. uniformis* and *Cx. vishnui* subgroup were highly abundant in the study areas similar to previous study conducted by Khan et al. [21]. These species happen to be established JEV vectors as well, which may explain the concurrent outbreaks of WNV and JEV in this region. An earlier study has also evidenced coinfection of both WNV and JEV in humans from this region during outbreaks in 2007 [15].

As sentinel chickens do not produce transmissible viremia, molecular analysis was not performed for the sampled chicken sera. However, molecular analysis of mosquito pools revealed WNV in *Ma. uniformis*, *Cx. vishnui*, *Cx. tritaeniorhynchus*, *Cx. quinquefasciatus*, *Cx. pseudovishnui* and *Cx. whitmorei.* The role of *Culex* species, especially *Cx. quinquefasciatus* have been well documented and implicated in WNV transmission in North America, Europe, South Africa and Australia [22]. In India, WNV has been isolated from *Cx. vishnui* during 1955–1958 and 1980–1981, and from *Cx. whitmorei* and *Cx. tritaeniorhynchus* in 1980. *Mansonia uniformis* have been found to carry WNV in Madagascar and Ethiopia [23, 24]. However, our study describes the first report of *Ma. uniformis* as a potential WNV vector in India. On the other hand, field-collected *Cx. pseudovishnui* has never been incriminated for WNV infection, although, a few experimental studies implied it to be a candidate WNV vector [25, 26]. Our study provides evidence that *Cx. pseudovishnui* is a potential vector for WNV transmission in this region. The incriminated mosquito species in our study are known as zoo-anthropogenic [27, 28]. Blood meal analysis of *Cx. quinquefasciatus* has demonstrated it to be highly ornithophilic besides feeding on mammals [29]. It is most likely that that this vector acts as an important bridge vector and aid in viral amplification.

Phylogenetic analysis revealed that all the sequences belonging to WNV Lineage 5 are similar to earlier report of WNV from Assam, India [11]. Based on isolates obtained from mosquitoes and humans in previous studies, there has been a prominent circulation of WNV Lineage 5 in India since its first detection in 1955 [30]. In our study, a distinct clade formed between the sequences from mosquitoes (2014–2015) and a WNV isolate from humans (2007) in Assam. This indicates a local circulation of this particular strain of Lineage 5 WNV in the given situation. However, the implications of synonymous and non-synonymous mutations observed in WNV strain in mosquito species require further studies.

The hemagglutination inhibition (HI) test used in this study has been considered as one of the standards for measuring neutralizing antibody titre. Previous studies have demonstrated the test to be specific as well as sensitive which is comparable to micro virus neutralization test [31]. Serological evidence of WNV infection in wild resident birds has been previously observed in Northern and Eastern India [32]. In this study, WNV antibodies were observed in sentinel chickens during the monsoon season: May-July, which is conducive for increase of potential vectors in this region. Although seroconverted chickens were not replaced with naïve ones and a low number of sentinel chicken were considered in this study, it indicated local WNV transmission that may be employed as an indicator of WNV activity in this region. The use of sentinel chicken to monitor WNV activity has been widely incorporated in many countries with WNV transmission including USA [33]. However, the involvement of other domestic birds of this region needs to be further investigated as earlier reports have shown WNV seroprevalence in domestic ducks and turkey [24, 34]. Wild migratory birds are often seen in this region, visiting water bodies and the banks of Brahmaputra, which is 10–15 km from all the study sites. Future studies involving sampling from migratory and other domestic birds may elucidate their role in the WNV transmission in this region.

## Conclusion

Our study found two new potential WNV vectors that could be playing a crucial role in WNV transmission in India. Detailed virus transmission studies as well as blood meal analysis of incriminated mosquito vectors would provide more evidence of the WNV cycle. Additionally, we corroborated that sentinel chickens can be useful to monitor WNV activity. These findings expand our understanding on vector diversity of WNV and help expedite future studies on the eco-epidemiology of WNV transmission.

## Additional file

Additional file 1: Table S1. Nucleotide divergence among WNV Lineage 5 sequences. (XLSX 10 kb)

## Abbreviations

HA: Hemagglutination
HI: Hemagglutination inhibition
JEV: Japanese encephalitis virus
ML: Maximum likelihood
NS1: Non-structural 1
RBC: Red blood corpuscles
RT-PCR: Reverse transcriptase polymerase chain reaction
WNV: West Nile virus
